# Gluteal Contractions as a Gateway to Sacral Plexus

**DOI:** 10.7759/cureus.21041

**Published:** 2022-01-09

**Authors:** Sandeep Diwan, Abhijit Nair, Bharati Adhye, Parag K Sancheti

**Affiliations:** 1 Anaesthesiology, Sancheti Institute for Orthopaedics and Rehabilitation, Pune, IND; 2 Anaesthesiology, Ibra Hospital, Ibra, OMN; 3 Orthopaedics and Trauma, Sancheti Institute for Orthopaedics and Rehabilitation, Pune, IND

**Keywords:** nerve block, neurostimulation, regional anesthesia, lumbar plexus, sacral plexus

## Abstract

Background

Neurostimulation-guided sacral plexus blocks (SPBs) are primarily indicated for surgeries of the foot and ankle and secondarily for supplementing a lumbar plexus block for hip surgeries. Although ultrasound has largely replaced neurostimulation-guided SPB, it may not be available at all facilities. Hence, it is prudent to understand the intricacies of neurostimulation-guided SPB.

Methodology

In this study, 10 American Society of Anesthesiologists-physical status I and II patients undergoing an intramedullary femoral nail procedure for femoral fractures of the shaft femur received a combined lumbar and sacral plexus block for operative surgery and postoperative pain relief. Neurostimulation-guided SPB was administered in all patients after the lumbar plexus block.

Results

In all patients, gluteal contractions were observed as the stimulating needle advanced during neurostimulation-guided SPB, which was either a dorsal or plantar flexion. The end-point of neurostimulation-guided SPB was obtained at 1-1.5 mm beyond the gluteal contractions.

Conclusions

It is important to understand that gluteal contractions are evident as the needle is advanced and can be considered a gateway during a neurostimulation-guided SPB.

## Introduction

Introduced by Mansour [1], a posterior approach to sacral plexus block (SPB) is indicated for foot and ankle surgeries with lumbar plexus or a femoral nerve as supplemental blocks. Although the point of needle insertion based on anatomical landmarks has been described, the depth of needle insertion remains elusive. The transverse process forms an important osseous anatomical landmark for the lumbar plexus block [2]. The distance between the transverse process to the lumbar plexus is 1.8-2.2 cm and is predictable, allowing a safe needle tip placement. However, a similar landmark does not exist for neurostimulation-guided SPB; though after encountering bone, a depth of less than 2 cm is recommended. Vital vascular and intestinal structures exist beneath the sacral plexus in the retroperitoneal area and demand cautious needle tip placement. Deep to the gluteus muscle is located the sacral plexus and its branches as it exits from the greater sciatic notch [3]. With continuous neurostimulation, gluteal contractions were perceived, which we thought was an important end-point to predict the depth of the sacral plexus.

In an embalmed cadaver in the prone position, an open dissection was performed to understand the relation of the origin of the inferior gluteal nerve (IGN) and its relationship with the sacral plexus. In the embalmed cadaver, cross-sections were performed at the level of the greater sciatic notch to appreciate the relation of sacral plexus with IGN and the depth of the sacral plexus from the pelvic peritoneum.

## Materials and methods

This study was approved by the Institutional Ethics Committee (dated March 11, 2021, Institutional Ethics Committee, Sancheti Hospital for Orthopaedics and Rehabilitation, Pune, India). A total of 10 American Society of Anesthesiologists-physical status (ASA-PS) I and II patients underwent an intramedullary nail for fractures of the shaft femur. Consent for procedures, using images without revealing the identity, and publication in a scientific journal was obtained from all patients. In a lateral decubitus position, a 100 mm stimulating needle was introduced (Stimuplex®, B. Braun Medical, Inc., Bethlehem, PA, USA) at Winnie’s anatomical landmark (lines passing through the posterior superior iliac spine and iliac crest intersected at one point which was the point of needle insertion), and lumbar plexus block was performed with the aid of neurostimulation. Following this, SPB was administered.

**Figure 1:**
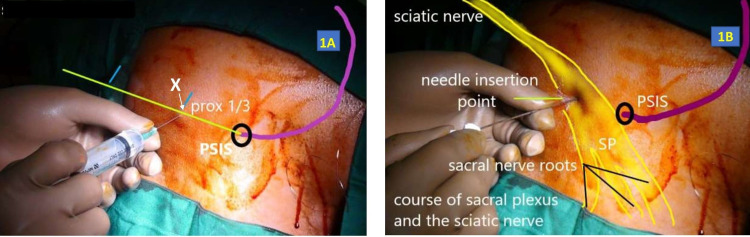
Sacral plexus block. A: Local anesthetic infiltration at point “X” on the line joining the posterior superior iliac spine and the ischial tuberosity. B: Stimulating needle inserted at the point “X.”

The anatomical landmarks for the posterior approach to the sacral plexus were a line joining the posterior superior iliac spine and the ischial tuberosity. This line was divided into proximal one-third and distal two-thirds. A point “X” was selected at the proximal one-third, and 1% xylocaine 5 mL was injected deep into the skin. With a 100 mm stimulating needle, the tip was advanced through the point “X” with continuous neurostimulation at 1.0 mA (Figures 1A, 1B). The path of the needle entry was through the skin, subcutaneous fat, and the gluteus maximus muscle. The direction of the needle was perpendicular to the skin at all times. The anticipated evoked motor responses were that of tibial nerve stimulation (plantar flexion) or common peroneal stimulation (dorsiflexion) of the foot.

## Results

The mean body weight of the patients was 60.4 kg. The needle tip encountered the transverse process of L4 in all patients during the lumbar plexus blocks. In 4/10 patients, erector muscle contractions were observed before the needle tip encountered the transverse process. Beyond the transverse process with an initial current of 1.0 mA, quadriceps contractions were evoked. The needle tip was positioned with a needle tip lumbar nerve contact at a current of not less than 0.4 mA. A 20-25 mL combination of equal proportion of 2.0% lidocaine and 0.5% bupivacaine was injected.

During the SPB, with the gradual advancement through the gluteal muscle, gluteal muscle contractions were obtained at 1.0 mA current at variable depths from the skin in all patients (10/10). The contractions disappeared when the current was lowered to 0.4 mA. Advancing the needle tip slowly did not evoke gluteal muscle contractions. However, after appreciating a loss of resistance in the form of a “click” led to an evoked motor response of the ankle joint and the foot, either a plantar or dorsiflexion with an increase in the current from 0.4 to 0.8 mA. This confirmed that the needle tip was close to the sacral plexus. The evoked motor response was fine-tuned at a current not less than 0.4 mA. Injection of 20 mL of a combination of equal proportion of 2.0% xylocaine and 0.5% bupivacaine led to the disappearance of muscle contractions (Video 1). The distance of the needle tip from obtaining the gluteal contractions to evoking the foot and ankle movements ranged from 0.5 mm to 1.5 mm. No bone was encountered during the SPB. No redirection and reinsertions of the needle were required.

**Video 1:** Video demonstrating gluteal contractions. The video demonstrates gluteal contractions during a neurostimulation-assisted sacral plexus block. The needle was then manipulated to get closer to the sacral plexus, which was confirmed by dorsiflexion of the foot.

**Figure 2:**
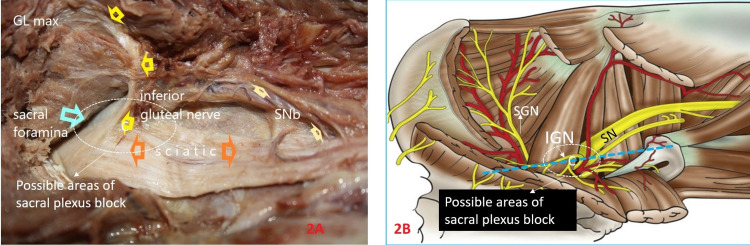
Images showing the origin of the inferior gluteal nerve. A: Prone cadaver dissection depicting the origin of the right inferior gluteal nerve (yellow hollow arrows) from dorsal of the right sacral plexus. The dotted oval white circle demonstrates the possible area of needle tip location; the light blue arrow illustrates the greater sciatic notch from which the sacral plexus arises and forms the sciatic nerve; the light yellow small arrows are the muscular branches from the sciatic nerve. B: Schematic illustrating the white dotted circle for possible needle tip location during sacral plexus block in the close vicinity of the inferior gluteal nerve. (Image source: Sandeep Diwan, Regional Nerve Blocks. 1st edition. Paras Publications; 2017. Sacral Plexus Block, Sandeep Diwan. pp. 257-61).

In an embalmed cadaver in the prone position, dissection confirmed that the origin of the IGN was dorsal to the sacral plexus. Before innervating the gluteus maximus, the IGN was juxtaposed with the sacral plexus as it emerged from the sciatic foramen (Figures 2A, 2B). The IGN then disappeared in the substance of the gluteus maximus muscle.

**Figure 3:**
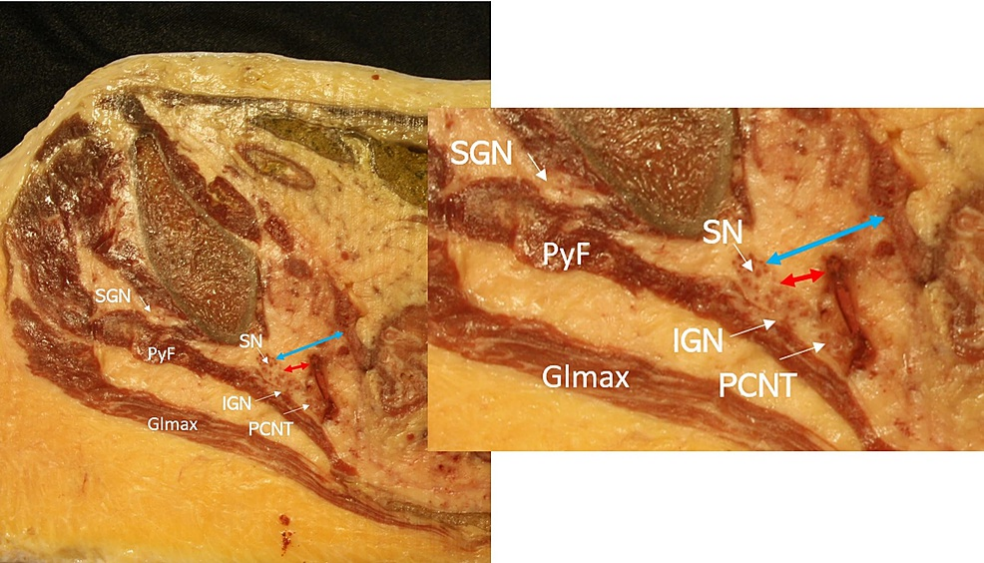
Cross-sectional view at the greater sciatic notch. Cross-section at the level of greater sciatic notch depicting the sacral plexus beneath the pyriformis. Between the GLamx and the GLmed is the superior gluteal nerve. Medial to the sacral plexus is the posterior cutaneous nerve, and ventral to the sacral plexus departing is the inferior gluteal nerve.

A cross-section at the greater sciatic notch (Figure 3) demonstrated the sacral plexus as it exited the greater sciatic notch, beneath pyriformis and in close association with the vascular and intestinal structures. The close relationship of the sacral plexus-sciatic nerve, the retroperitoneal, and the vascular structures are depicted in Figure 3. The superior gluteal nerve in the plane between the gluteus medius and maximus could be visualized. The posterior cutaneous nerve was medial to the sciatic nerve, and the IGN departing from the sacral plexus could be seen beneath the pyriformis. The inferior gluteal artery and the pelvic structures were observed at a distance from the sacral plexus.

## Discussion

The posterior approach to the sacral plexus was described by Mansour [1]. Being a true plexus block, it provides anesthesia in the distribution of a sciatic nerve, posterior cutaneous nerve of the thigh, and the articular branch to the hip joint [4]. With branches of the sacral plexus emerging at the level of the greater sciatic notch [5], needle tip placement at this point and local anesthetic injection would block the following nerves: superior (L4-S1) and inferior (L5-S2) gluteal nerves, pudendal nerve (S2-S4), nerve to the quadratus femoris and inferior gemellus muscles (L4-S1), nerve to the internal obturator and superior gemellus muscles (L5-S2), posterior femoral cutaneous nerve (S1-S3), and parasympathetic pelvic splanchnic nerves (S2-S4).

Definitive anatomical landmark exists for deeper blocks; the transverse process and the costotransverse junction for the lumbar plexus and thoracic paravertebral blocks, respectively [6]. The depth of the needle tip should be 1.8-2.2 cm in the lumbar plexus block and not more than 1 cm in the thoracic paravertebral block. However, in the SPB, after encountering the bone (a part of the ilium), the needle depth should be less than 2 cm. Variable success rates have been reported with Mansour’s technique, ranging between 94% and 66% [7,8]. The difference in the incidence may be due to different definitions of needle passes (reinsertion versus redirection).

According to a magnetic resonance imaging (MRI) anatomical study [9] of sacral plexus in volunteers, needle stimulation using Mansour’s approaches resulted in 50% encountering the sacral plexus. Overall, 30% of the time the needle penetrated either the small bowel, rectum, or vascular structure at first needle pass. With caudal reinsertion of the needle, the same study reported unwarranted needle tip placement in the rectum and perianal fat (40%). Further, MRI images [9] depicted that, after encountering the bone (ilium), limiting the needle tip depth to less than 2 cm as recommended was not favorable. Thus, we thought it was prudent to assess the possible depth of the needle tip to the sacral plexus.

MRI evaluation of sacral anatomy provides valuable information regarding its relationship with the sacral plexus [10]. At the level of the greater sciatic foramen, the sacral plexus is situated in close relationship with the pelvic viscera. Hence, it is advisable not to advance the needle beyond the gluteus maximus aimlessly.

The dorsal branches of the fifth lumbar and first and second sacral form the IGN [11]. The IGN leaves the pelvis via the greater sciatic foramen inferior to the piriformis and divides into at least four branches that pass posteriorly deep to the gluteus maximus [11]. The IGN is visualized close and medial to the sacral plexus and emerges beneath the pyriformis muscle [12,13]. With our needle insertion point for SPB, the IGN was in the needle trajectory as it approached the sacral plexus. Contractions of the gluteal muscle confirm the needle tip close to the IGN. Carefully advancing the needle to 0.5-2.0 mm, the tip would be adjacent to the sacral plexus. Although we do not deny the possibility of neurostimulation of one of the four motor branches of the IGN, these would be localized contractions of a specific area of the gluteal muscle. MRI at the level of the greater sciatic foramen can perceive the path of the IGN in the coronal plane; however, real-time detection of the IGN using ultrasound is questionable [14]. Nevertheless, a single case report illustrated the location of the superior gluteal nerve in the plane between the gluteus medius and minimus muscles close to pulsating the superior gluteal artery [15]. Comparable specificity (86%) was demonstrated when ultrasound was juxtaposed with MRI for the detection of peripheral nerve disorder; however, ultrasound had a greater sensitivity than MRI (93% vs. 67%) [16].

Based on our cadaveric findings and the proximity of IGN with the sacral plexus, neurostimulation of the IGN is possible, indicated as contractions of gluteus maximus muscle. Further, the distance between the inferior portion of the ilium might be encountered with the advancing needle tip, and the pelvic visceral can be less than 2 cm. Hence, an earlier recommendation of limiting the distance to less than 2 cm after encountering the bone (ilium) needs to be abandoned.

Immediate follow-up at discharge and after three months did not reveal neurological adverse effects regarding the weakness of the gluteus maximus and foot-ankle movements. Hence, contemplate the contractions of the gluteal muscle secondary to the neurostimulation of the IGN as the gateway to the sacral plexus. Conversely, it implies the possibility of impalement of IGN if gluteal contractions remain unrecognized during an anatomical landmark-guided SPB. We thought it is pertinent to put this forth for anesthesiologists who provide non-ultrasound, landmark-based neurostimulation-guided SPB.

There are several limitations of this study. It was a small case series, and ultrasound was not used for the real-time visualization of structures. The findings of this series need to be validated by comparative studies in the future.

## Conclusions

As IGN is in close relationship to the sacral plexus, the contraction of the gluteus maximus muscle on neurostimulation can be considered as the point of injection for a successful SPB. Further studies with an adequate sample size are needed to justify this approach. We also recommend comparing neurostimulation-guided SPB, as described in this study, with an ultrasound-guided block to compare the success rate and safety.
